# Perceptions of Deficiencies in the Basic Conditions for Farm Management and Quality of Life in Coffee‐Growing Households: A Panel Analysis of a Rural Community in Eastern Uganda

**DOI:** 10.1002/gch2.202300196

**Published:** 2024-03-12

**Authors:** Anna Lina Bartl

**Affiliations:** ^1^ Agricultural Economics and Rural Development University of Göttingen 37073 Göttingen Germany

**Keywords:** climate change, coffea arabica, constraints on quality of life, deficiencies in conditions for farming, knowledge accessibility, resource levels, Uganda

## Abstract

In the present study, information collected from 360 coffee‐cultivating households (HHs) is used to investigate perceptions of deficiencies in three sub‐counties in Eastern Uganda and to study changes in these perceptions between two survey rounds. The results of an explorative principal components analysis identify five factors affecting farmers’ perceptions. Whereas perceptions of deficiencies in the preconditions for farm management activities differ significantly between the three sub‐counties investigated, indicators of deficiencies in general life quality are distributed more equally. Deteriorations are explained mainly by perceived changes in weather conditions. On the one hand, it can be assumed that the high constraint level will continue to increase in the future due to climate change and its impacts on life quality and the basic conditions required for farm management. On the other hand, access to resources such as water taps but also increased competition between buyers, have improved the situation. Results further indicate that if activities such as the expansion of information access and improvement of road conditions (after land registration) are implemented on a larger scale, these negative trends can be partly counteracted to help farmers maintain the conditions for effective farm management and improve their quality of life in the future.

## Graphical abstract

Deteriorations in the conditions for coffee farming and quality of life are explained mainly by perceived changes in weather conditions. Access to material resources, intellectual resources, as well as increased competition between buyers, have improved the situation, resulting in less opportunistic behavior on the part of buyers as well as better quality and price awareness among farmers.

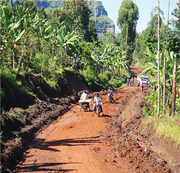

## 1 Introduction

It is well known that smallholder coffee farmers worldwide face numerous deficiencies in life quality and challenges in coffee production. They are often barely able to meet their most basic needs. According to,^[^ ^1^ ^]^ coffee yields are relatively low overall in Africa and sell at even lower prices than coffee from other continents.^[^ ^1^ ^]^

Recent data analyses show that 18.7 million Ugandans, or more than 42% of the population, were defined as poor in 2019 based on the international poverty line of 2.15 USD purchasing power parity per capita established in 2017.^[^ ^2^ ^]^ Also, the majority of smallholder coffee farmers in the Mount Elgon region are below the income threshold for extreme poverty and suffer both under‐ and malnutrition, deficiencies in health care, and many other needs. In addition to the lack of liquidity and infrastructure, farmers in this region, similar to other coffee farmers worldwide, often do not have access to the information and equipment needed for proper farming and face increased pest and disease incidence and abruptly changing coffee prices.^[^ ^3^, ^4^ ^]^Research of different coffee growing areas agrees that the wellbeing level and the relation between given challenges and resources, financially but also informational could be seen as major pre‐condition for sustainable production in coffee‐growing regions.^[^ ^5^, ^6^ ^]^ Research predicts that the wellbeing of coffee farmers in the eastern part of Uganda is deteriorating due to changing weather conditions and decreasing land per household.^[^ ^7^ ^]^ On top of that, it can be assumed that the increasing stressors in the ongoing global change are also impacting farming practices negatively in the Mount Elgon region as well, as Jezeer et al. found for coffee‐growing HHs in Peru.^[^ ^5^ ^]^ To develop strategies that help the farmers cope with present and future challenges and allow them to implement sustainable coffee management systems that are resilient and productive, research is needed on existing deficiencies and resource levels.

Agricultural systems are closely interlinked with farmers’ private lives. Coffee farmers usually live on their small farms, cultivate other foods to provide sustenance for their families, and use the same very limited resources for gardening and other household needs. When faced with decisions on investments, farmers are therefore often required to decide between meeting their families’ basic needs or improving the quality of their farming operations. As in Peru and other Latin American countries where research found that a lack of financial assets limits access to input, it is very likely that also in the here investigated area, wealthier farms are more likely to invest in sustainable farm management systems, especially when not facing a lack in basic needs.^[^ ^5^, ^8^ ^]^

Most of the research to date dealing with constraint levels and deficiencies does not study the preconditions for farming and general life quality, nor does it investigate the perceptions of farmers themselves. Most studies that do investigate life quality deal with specific topics and focus on even narrower subtopics;^[^ ^9^, ^10^, ^11^, ^12^ ^]^ are only a few such studies.

As also in other coffee‐growing regions, the Ugandan coffee farmers’ perceptions of constraints to coffee production and life quality have not been examined broadly enough to draw a complete picture, to rank the constraints farmers face, and to develop strategies addressing the deficiencies that present the greatest constraints. To address the gap in research on farmers’ perceptions of the deficiencies coffee‐producing HHs face in Uganda, the present paper identifies categories and levels of deficiencies in the conditions for coffee farming and in quality of life of coffee‐farming HHs. It is based on quantitative and qualitative answers from 360 smallholder coffee farmers in three sub‐counties in the Mount Elgon region who were interviewed in 2018 and 2019. Furthermore, it analyzes and discusses changes in perceptions deriving from a comparison between the two survey rounds.

This paper uses an explorative principal component analysis (PCA) to classify the indicators. A one‐factor ANOVA and a t‐test are used to test the hypotheses that (1) deficiency perceptions are not distributed equally within and between sub‐counties in the Mount Elgon region and across the two survey rounds. The paper further investigates farmers’ qualitative responses to test the second hypothesis that (2) climate change and other externally influenced conditions have an impact on the perceived constraint level.

## 2 Experimental Section

### 2.1 Data Collection

Data were collected as part of the project “Potential improvements for the income situation of smallholder coffee farmers in Mount Elgon, Uganda” developed and implemented by the Georg‐August University of Göttingen, Germany, and the National Agricultural Research Organization (NARO) of Uganda. Questionnaire pre‐tests were developed and implemented in the area to evaluate the feasibility of interviewing and to test content and construct validity and reliability.

### 2.2 Study Area

The study was conducted on the Western slopes of the Mount Elgon region, one of the three main Arabica coffee–producing regions in Uganda, where, for many farmers, Arabica coffee cultivation is the main source of income. It is estimated that 90% of coffee cultivation takes place on plots of less than three hectares, with a trend towards smaller plots.^[^ ^13^ ^]^

**Figure 1:**
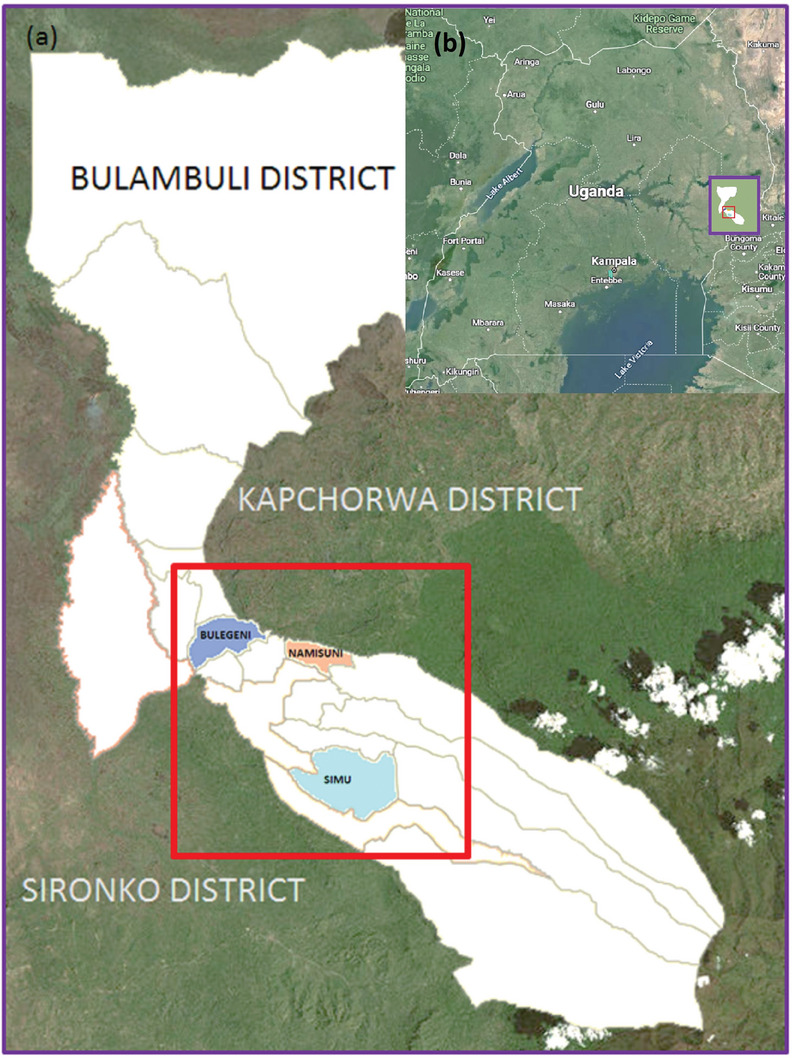
Map of a) South Uganda and b) details of Bulambuli district with Bulambuli County (grey) and Elgon County (white) with the sub‐counties Bulegeni (blue), Simu (turquoise), and Namisuni (orange)^[^ ^7^ ^]^ here (*n* = 360).

Data collection for this study took place in the Bulambuli district, which extends over about 809 km^2^, covers elevations ranging from 1400 to over 1700 meters above sea level, and is divided into two counties, Elgon and Bulambuli County.^[^ ^14^ ^]^ Surveys were conducted in Elgon County because 60.5% of its HHs are engaged in coffee growing, whereas in Bulambuli County, only 2.2% of existing HHs are coffee farmers.^[^ ^14^ ^]^ In Elgon County, the three sub‐counties of Bulegeni, Simu, and Namisuni were chosen (**Figure** **1**).

The first round of the panel survey took place from July to December 2018 in the three sub‐counties of Elgon County. In a second round from September 2019 to January 2020, HHs from the previous survey round were interviewed for a second time to identify changes between the two survey rounds. The periods for survey rounds were aligned with the coffee harvest season to enable better comparison of conditions for the HHs between two seasons.

From 460 randomly chosen coffee‐cultivating HHs, 29 did not provide (sufficiently good) data, which led to a final data set of completed questionnaires from 431 HHs (**Table** **1**). In 2019, 360 of the previously selected 431 HHs could be reached for another interview: 134 HHs from the sub‐county of Bulegeni, 77 HHs from Simu, and 149 HHs from Namisuni.

**Table 1:** Sample demographics (*n* = 360)

Quantitative data set		Bulegeni	Simu	Namisuni	Total	Research area
Number of HHs		*n* = 134	*n* = 77	*n* = 149	*n* = 360	21244^a)^
Gender of HH head	Male	94,0%	93.5%	95,3%	94.4%	81.4%^a)^
	Female	6.0%	6.5%	4.7%	5.6%	18.6%^a)^
Age of HH head	<18	0.0%	0.0%	0.0%	0.0%	1.0%^a)^
	18‐30	6.0%	11.7%	16.4%	11,5%	25.9%^a)^
	31‐59	59.7%	62.3%	65.8%	62,7%	53.9%^a)^
	>60	34.3%	26.0%	17.8%	25.8%	19.2%^a)^
Highest level of education for head of HH	Illiterate	4.6%	5.2%	2.1%	3.7%	9.3%^b)^
	Primary school	44.3%	41.6%	61.0%	50.6%	58.7%^b)^, ^c)^
	High school	45.8%	46.8%	33.6%	41.0%	27.8%^b)^, ^d)^
	College	4.6%	2.6%	2.7%	3.4%	8.2%^b)^, ^e)^
	University	0.8%	3.9%	0.7%	1.4%	
People per HH	MD/SD	6.38/2.393	6.40/2.273	5.30/2.092	5.94/2.303	4.638/0.135^b)^
Coffee production is the main source of income		83.5%	92.2%	87.9%	87.2%	83.0% major economic activity is crop farming^b)^

^a)^ Data for Elgon County^[^ ^9^ ^]^;

^b)^ Data for Elgon Region^[^ ^15^ ^]^;

^c)^ Sum from category: some primary and completed primary for the whole HH.

^d)^ Sum from category some secondary and completed secondary for the whole HH.

^e)^ Post‐secondary and above for the whole HH.

### 2.3 Sample Description

The sample characteristics of the HHs interviewed in both survey rounds are depicted in Table 1 and used for data interpretation only. Comparing the sample distribution with the average HH characteristics for the area in which the study was conducted, a slight deviation in socio‐demographic parameters can be seen.

### 2.4 Statistical Analysis

The pre‐processing of data was done with pre‐tests implementation before survey, data cleaning by, e.g., elimination of interviews was showing low data quality, testing of final data set for normal distribution. Data presentation are shown, e.g., in the Supporting Information section presenting Mean, SD, SE. The sample size was for all tests *n* = 360. Statistical methods used were: Principal component analysis (PCA) to classify the indicators. A one‐factor ANOVA and a *t*‐test are used to test the impact of time and sub‐county on the perception of deficiency. Details of each test could be found in and below the tables in the Supporting Information Section. IBM SPSS Version 27 was used for statistical analysis.

## 3 Results

Farmers were interviewed about their perceptions of deficiencies in the basic preconditions for farm management activities and general life quality. They were asked to rate 16 deficiencies on a scale from 1 (no constraint) to 5 (major constraint). The indicators were classified into the basic preconditions for farm management activities (10 items) and general life quality (6 items).

The results of the survey show that the mean constraint level largely exceeds 3.0, indicating that farmers felt “more or less constrained” in at least one of the two survey rounds. For most items, the mean in constraint levels was above 4 in 2018; only *lack of insurance*, *distance to a water source*, and *water quality* showed means below 4. From the first survey round in 2018 to the second in 2019, constraint levels except *bad roads*, *lack of health care nearby*, *lack of insurance*, and *poor infrastructure* declined. However, the standard deviation for all variables except for *bad roads*, *water quality*, and *water source*, increased for the whole sample. In Bulegeni, the change in standard deviation was smaller in both years.

Along with the quantitative figures from the scales, the survey also asked farmers whether they perceived a change in deficiency levels between 2018 and 2019. If the farmers perceived changes between the two years, they were asked to specify how the situation had improved or deteriorated for the specific item.

Two thirds of the farmers in the samples perceived that the situation had either deteriorated overall (35.18%) or that it had improved for some indicators and deteriorated for others (29.64%). The other third of the farmers either perceived no change (18.56%) or felt that the situation had improved (16.62%). The percentage of farmers who perceived no change between the two survey rounds was higher, and the percentage who saw an improvement in some indicators and a deterioration in others was lower in Bulegeni than in the two other sub‐counties.

**Figure 2:**
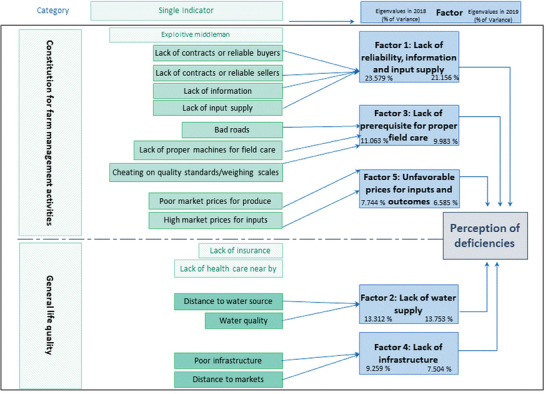
Summarizing the components of the perception of deficiencies, the indicators investigated and the results of the PCA survey rounds in 2018 and 2019, *n* = 360.

To enable a well‐structured interpretation of results, a PCA was applied to the 16 deficiency variables. The best result of the PCA (shown in **Figure** **2**) was found for a five‐factor solution, explaining 57.807% of the total variance in 2018 and 58.982% of the total variance in 2019 (Kaiser‐Meyer‐Olkin Measure (KMO) = 0.667 in 2018 and KMO = 0.695 in 2019) by including 13 of the previously derived indicators.

When investigating the impact of time and sub‐county on these factors, no significant difference between the two survey rounds could be found for any of the factors. In contrast, the ANOVA found a significant difference between sub‐counties for Factor 1, Factor 2, and Factor 3 in 2018, and one coffee harvest season later, for all factors except Factor 1 (see tables in the Supporting Information section). The results require further investigation with regard to the classifications and factors. Details of the means and SD for the five factors belonging to the basic preconditions for farm management can be found in the Supporting Information section (Table S1, Supporting Information).

### 3.1 Preconditions for Farm Management Activities

In the preconditions for farm management activities group, the greatest improvement from 2018 to 2019 was found for Factor 1, *lack of reliability, information, and input supply*. To gain better insights into the perceived limitation in the daily life of the farmers, each factor is examined individually in the following section.

Factor 1 consists of a *lack of contracts with reliable buyers*, *lack of contracts with reliable sellers*, *lack of information*, and *lack of input supply*. The t‐test showed a highly significant impact of time on these four variables (*P* = 0.000***); details can be found in Table S1 (Supporting Information). For all of the aforementioned indicators, the total mean of constraint levels declined from 2018 to 2019 both for the whole sample and also when looking at the single sub‐counties: For *lack of reliable buyers* 4.13 ± 0.89 in 2018 and 3.24 ± 1.17 in 2019, for *lack of reliable sellers* 4.08 ± 0.93 in 2018 and 3.22 ± 1.13 in 2019, for *lack of information* 4.11 ± 0.88 in 2018 and 2.97 ± in 1.31, and for *lack of input supply* 4.15 ± 0.91 in 2018 and 3.42 ± 0.93 in 2019.

Five of the farmers interviewed mentioned improvements in contracts with buyers and reliability of buyers. One commented that “buyers are more reliable and more willing to buy our produce.” Two farmers saw an increasing number of companies coming into the area “to buy produce from farmers like Kawacom and signing contracts with them.” Interestingly, most of the farmers did not switch buyers from 2018 to 2019 even though the diversity of choices increased. Also, the percentage of farmers selling to more than one buyer remained the same; in 2018 and 2019 only 5% of the interviewed HHs divided their harvest. As the reason for the improvement, two farmers mentioned that they now “have a right to search for higher prices for their produce/coffee.” Access to information also seems to play a role in the reduction of constraint levels for this factor, which is evident in all sub‐counties for the item *lack of information*. For 12 HHs, the constraint level *lack of information* declined from 2018 to 2019. An increase in information sources was cited as a reason for this: Farmers perceived themselves as having better access to information, “for example, from radios, other farmers, and even the market” and “access to extension services and access to groups.” Here, the radio seems to be the most trusted source of information. One farmer mentioned that there was an improvement of information access “thanks to the many radio programs, but it is up to me to decide on what to do.” Four of the 12 HHs noted that the improved access to information provides orientation concerning coffee and other prices of products. One farmer said that “We have enough sources of information to get better prices to improve people's living standards.” In addition, it was mentioned that “the information from technical staff, such as community development officers, has improved agricultural production.” The increasing information on agricultural production also has an impact on the improvement of perceived input supply. The “extension of services for organic farming in the sub‐county of (Simu)” and the fact that “the majority use organic fertilizers like chicken droppings and cow dung” were mentioned as reasons for better input supply as compared to one year ago.

Three HHs perceived the situation to be worse than in 2018. They noted that there were no contracts for coffee‐selling activities because “the lack of contract buyers causes prices of coffee to fluctuate every year.” Another farmer in the same sub‐county agreed, saying “It is better to enter into a contract with buyers at agreed prices, which we pay attention to.” Another farmer mentioned that “there are fewer reliable buyers and sellers because of the bad condition of the roads.”

The ANOVA found no significant impact of the sub‐county on *lack of information* or *lack of input supply* in 2018. In contrast, highly significant (*P* = 0.000***) results for the impact of sub‐county on *lack of reliable buyers* and *lack of reliable sellers* were found in 2018. The pairwise comparison found only significant results (*P* ≤ 0.05*) for the difference between Simu (4.28 ± 0.65) and Bulegeni (3.76 ± 1.24) and between Namisuni (4.27 ± 0.59) and Bulegeni for a *lack of reliable sellers* in the same year. The ANOVA showed a highly significant impact of sub‐county on all four deficiency items belonging to Factor 1 in 2019. There, the difference between Namisuni and Bulegeni was highly significant (*P* ≤ 0.001***) for all variables, showing in the second year for all variables higher means of constraint levels in Bulegeni than in Namisuni. A significant (*P* ≤ 0.050*) difference between Simu and Bulegeni could be found for *lack of information* and *lack of input supply*, also showing a higher mean in constraint levels in Bulegeni than in Simu. The results for Simu and Namisuni only differed for the *lack of reliable buyers* (*P* = 0.023*), which showed a lower mean constraint level in Namisuni.

Factor 3 consists of *bad roads*, *lack of proper machines for field management*, and *cheating on quality standards/weighing scales*. The *t*‐test showed a highly significant impact of time on all the indicators (*P* = 0.000***). The perceived constraint level of *bad roads* increased for the whole sample from 4.58 ± 0.73 in 2018 to 4.79 ± 0.56 in 2019; the same trend could be seen for each sub‐county. For the perception of a *lack of proper machines for field care*, the constraint level decreased from a total mean of 4.37 ± 0.75 in 2018 to 4.07 ± 0.80 in 2019. Also, for *cheating on quality standards/weighing scales*, the means decreased from 4.58 ± 0.56 to 3.95 ± 1.33 for the whole sample as well as for each sub‐county.

One hundred eighty‐four farmers gave qualitative responses and described the perceived deterioration of road conditions. All of them mentioned that “the roads are in bad condition (…) when it rains, we are unable to move,” and “it is always muddy and dusty.” Many of them cited climate change and the heavy rainfalls in the area as reasons for the bad roads. Although eleven of those farmers mentioned that “the roads are bad due to lack of support from the (local) government,” most of the farmers perceived the (changing) weather to be the reason for the bad roads or mentioned that “road conditions are bad especially after it rains”: In total, 98 farmers mentioned that the weather or climate change negatively affects transport: “Heavy rainfalls lead to flooding, affecting our roads and hindering transport.” Of those 98 farmers, 40 mentioned that the conditions of roads are becoming worse due to “negative changes in the climate, which have resulted in heavy rainfall.” The bad roads not only “hinder transport of agricultural products” and “thus affect production and produce”; “they have also made transport expensive,” which is “leading to low income.” Only eleven farmers mentioned economic losses due to bad road conditions explicitly, two of them located in Simu. Many others addressed the economic impacts indirectly, for instance, with comments like “very bad roads [that] have hindered transportation of produce to better markets hence selling locally.” Other farmers claimed that the “poor roads scare people into moving because they lack transport,” “poor roads put [our] lives at risk,” “the condition of roads even causes accidents,” and the “poor roads have made it difficult to access proper health care.”

The perceived improvement in Factor 3, *lack of prerequisites for proper field care*, came in large part from reduced cheating on quality standards/weighing scales: 18 HHs perceived an improvement in this area. Six farmers who perceived an improvement explained it through their awareness of coffee quality: “We are aware of coffee quality so we can't be cheated.” Another farmer mentioned “We now have enough information about coffee, and we have been trained by the sub‐county official on how we can produce good quality coffee,” three other farmers agreed. They further added “we have acquired skills in processing coffee and therefore now produce good‐quality coffee” and “we know the required standards for coffee, which increases our income too,” “we are not cheated because we set our own prices” and “now we know the required standards/quality of coffee.” Another reason mentioned for the improvement of this indicator was again that farmers “have enough information” and “there is transparency and accountability at the district level.” In addition, seven of the HHs that perceived an improvement explained that “the weighing scales are now on standard” because the “government has ordered every businessperson to take their weighing scale for stamping” (that is, for calibration and verification). Four of these HHs perceived an improvement “due to possession of my personal weighing scale.”

However, even for indicators where mean constraint levels declined across the two survey rounds, some farmers mentioned and provided qualitative explanations for a perceived deterioration of conditions. Ten farmers perceived the current situation to be worse than in one of 12 preceding months. Six of those farmers made comments such as “most buyers cheat farmers on the quality standards [for] coffee and the weighing scales they use” because they “cannot really tell if the weighing scale is good or not,” and said that they had experienced “much cheating on coffee quality by buyers.”

Five farmers saw an increase in constraint levels for the item *lack of proper machines for field management*. They claimed that there was “a huge increase in agricultural machines like tractors every year” but that “due to inaccessibility” and “increased poverty,” only other farmers were able to take advantage of the “increase in hiring and buying of machines (Simu).”

The results of the ANOVA showed no significant impact of the sub‐county on any of the indicators in 2018 (*P* ≥ 0.050), but a very highly significant (*P* = 0.000***) impact of the sub‐county on the item *bad roads* in 2019. The pairwise comparison for the item *bad roads* showed a highly significant difference (*P* = 0.000***) between Simu (4.90 ± 0.34) and Bulegeni (4.62 ± 0.70) and between Namisuni (4.88 ± 0.47) and Bulegeni, but no significant difference between Simu and Namisuni for that item in 2019. For the item *lack of proper machines for field care*, results also showed a significant difference between Namisuni (4.00 ± 0.72) and Bulegeni (4.20 ± 0.87) in 2019, but on a lower significance level (*P* = 0.011*).

Factor 5 consists of the items *poor market prices for products* and *high market price for input*. The *t*‐test indicated a highly significant (*P* = 0.000***) decrease in constraint levels between the two years for the two items belonging to this factor: for *poor market prices for produce* from 4.43 ± 0.66 in 2018 to 3.91 ± 0.84 in 2019, and for *high market price for input* from 4.45 ± 0.66 to 3.86 ± 0.97.

Of the farmers who gave a qualitative response for that factor, 18 perceived an improvement. They noted an increase in the price of products and mentioned that “the price of coffee has improved.” One farmer commented “I have achieved a much greater harvest this year and with a better market price.” Four farmers from all sub‐counties explained “we have gained skills in that we can store our produce until prices rise and seek better prices.” In addition, “companies (…) have improved on prices of coffee” and the “extension services by NGOs (…) have led to the use [of] organic fertilizer to avoid high prices of fertilizer,” resulting in the fact that “income and even the way of life has changed” for those farmers.

Although the means decreased over time, 34 farmers mentioned perceiving the situation to be worse than one year earlier. Across all sub‐counties, seven farmers attributed the deterioration to bad roads: “due to climate change, which has affected the conditions of most roads in the area, the market for products is deteriorating,” and because of the impassable roads, farmers said that they “could not save their produce in better markets, so they sell locally at poor prices.” Another added that “it becomes expensive to transport the harvested coffee to better markets.” In general, “the input cost is high compared to output, hence creating losses.” One farmer said “I borrowed money (got a loan) to finance my input, but the yield has not met my expectations, hence I have made losses.” Another added “I am putting a lot of money into inputs like fertilizers, pesticides, etc., so making losses.” In addition, “the increase of pests and diseases affecting the crops in the area is leading to poor quality [produce], which is causing the market to deteriorate.” Other factors impacting the poor market prices for products were “…too much sun,” “severe drought,” and resulting “low productivity.” The “poor” and “unpredictable market prices, which change abruptly, affect us greatly, thus causing losses”^[^ ^16^ ^]^ were also mentioned as the reason for the deterioration of this factor. In addition to the “price fluctuation due to bumper harvest prices,” the “fluctuation of our currency” was mentioned as affecting farmers’ income negatively.

The one‐way ANOVA did not find any impact of sub‐county on any of the constraint groups in 2018. In 2019, however, it showed a significant (*P* = 0.029*) result for the impact of sub‐county on high market prices for inputs, but with no significant result for any pairwise sub‐county comparison after correction.

The constraint level for an *exploitative middleman* could not be included in any of the five factors and was investigated individually. According to the results from the *t*‐test, the impact of time on this constraint indicator is highly significant (*P* = 0.000***), showing a decrease in perception of deficiency for an *exploitative middleman* from a total mean of 4.39 ± 0.71 in 2018 to 4.01 ± 0.99 in 2019.

Eleven farmers explained why they were less constrained by the item *exploitative middleman* than in the previous year. One explanation was that “farmers have access to information and can now look for high prices and have also acquired skills in processing coffee so they cannot be exploited.” The other explanation was that “many companies have come, thus eliminating middleman exploitation and resulting in buyers paying higher coffee prices.”

However, even if the constraint level on *exploitative middlemen* decreased over the two survey rounds, the means were also very high in 2019, so it is unsurprising that farmers answered open‐ended survey questions by expressing dissatisfaction or explaining why they perceived the situation to be worsening. Five farmers reported a more *exploitative middleman* than in the previous harvest season. One saw an “increase in the number of middlemen in the area”; others claimed “the buyers cheat farmers a lot, and this has reduced income” or that buyers “hide good prices from us farmers” and that “most of them are thieves” or that “we (farmers) share the view that if (a buyer) refuses to agree with us, we cannot sell to them.”

In 2018, a highly significant difference (*P* = 0.007**) between sub‐counties was only found between Simu (4.26 ± 0.66) and Bulegeni (4.47 ± 0.79). The result for the impact of sub‐county was only given for the Kruskal‐Wallis test (*P* = 0.007**), the ANOVA performed previously showed no significant result (*P* = 0.113).

In 2019, no significant impact of sub‐county on the perceived deficiency of exploitative middlemen could be found (P = 0.111).

### 3.2 General Life Quality

The general life quality group includes Factor 2, *water supply*, and Factor 4, *infrastructure*. The single items of *lack of insurance* and *lack of health care nearby* could also be grouped into general life quality and interpreted further.

The deficiency indicators for the general life quality indicators show the lowest constraint level but at the same time the highest standard deviation (up to 1.78) for *distance to the water source* (2.05 to 3.15) and *water quality* (3.01 to 3.54). *Lack of health care nearby* and *distance to markets* show the highest means for constraint level in the general life quality indicator group (up to 4.57), whereas the *lack of insurance* and *poor infrastructure* are perceived as having a less negative impact on quality of life (3.58 to 4.28).

Factor 2, *water supply*, consists of *distance to the water source* and the constraint level on *water quality*. In 2018, the factor *lack of water supply*, comprising the variables *water quality* and *distance to water source*, had the lowest level of constraint overall (3.004) as well as in Namisuni (2.686), Simu (3.074), and Bulegeni (3.343). Following the paired t‐test, the impact of time on the *distance to the water source* was highly significant (*P* = 0.002**): The mean perceived constraint level was lower in 2019 than in 2018: *Distance to the water source* decreased from 2.94 ± 1.72 in 2018 to 2.56 ± 1.58 in 2019 and *water quality* was perceived in total means of 3.10 ± 1.68 in 2018 and 3.01 ± 1.53 in 2019.

Ninety‐five of the interviewed HHs mentioned seeing an improvement in the *water supply*. Of these, 81 HHs explained the improvement in the “distribution of water taps” and that the “extension of piped water by the government” “solved the lack of water” or “shortened the distance.” Along with the water availability nearby, the improvement was also linked to perceived better water quality: “We now have access to clean drinking water, that is to say, piped water” “which is already treated.” However, two farmers mentioned that the tapped water “has to be paid for monthly.”

Four others mentioned the “water protection provided by the government to every homestead.” One farmer mentioned heavy rains as the reason for better water availability.

However, nine farmers mentioned experiencing the *water supply* to be worse than one year earlier. Three mentioned that “the distance to the water source has deteriorated.” One added that the “long distance to the water source has affected my agro‐production since it is not easy to get water to irrigate crops.” For the others that mentioned a deterioration in the *water supply*, the major reason was a decrease in *water quality*: “the water is somehow clean during the dry season but dirty during the rainy season.” Two others agreed, stating “the water is very dirty, especially when it rains.”

The one‐factor ANOVA performed for data collected in 2018 showed a very high significant (*P* = 0.000***) impact of sub‐county on the perception of *water quality*: A very high significant level (*P* = 0.000***) was found for the difference between Namisuni (2.74 ± 1.70) and Bulegeni (3.54 ± 1.53) in 2018. In 2019, both *distance to water source* and *water quality* were significantly impacted by sub‐county (*P* = 0.000***). The constraint level for *distance to the water source* differed highly significantly (*P* ≤ 0.001***) between Simu (2.84 ± 1.60) and Namisuni (2.05 ± 1.41) and between Namisuni and Bulegeni (2.96 ± 1.61). For the item *water quality*, a similar trend was evident, except that in this case, farmers located in Simu, followed by Bulegeni, perceived the highest constraint level. Farmers in Simu (3.44 ± 1.40) and Namisuni (2.63 ± 1.50) perceived the constraint level for *water quality* differently from farmers in Bulegeni (3.16 ± 1.55). No difference between Simu and Bulegeni could be found.

Factor 4 includes the constraint level perception for *poor infrastructure* and *distance to markets*. A significant impact of time was only found for the reduced constraint level for *distance to markets*. For *distance to markets*, the change in total mean from 4.47 ± 0.77 in 2018 to 4.35 ± 0.82 was only significant when applying the Wilcoxon Test (*P* = 0.020*), not for the results shown by the performed t‐test.

An improvement in the perception of Factor 4 was explained by six HHs located in Bulegeni. Two farmers mentioned that the *distance to markets* constrained them less than one year ago, because “the local traders even come up to the home to buy the coffee” and because the farmers could “sell anywhere to thus avoid the middleman.” One farmer added that “life has changed because of modernization of agriculture, especially coffee,” two others mentioned higher production, and one of them mentioned that “knowledge and skills in bookkeeping boost production.” Another commented that “life has improved because of good produce transport.” No farmer perceived a deterioration due to the factor *infrastructure*.

However, the investigation of the impact of the sub‐county on the indicators resulted in a highly significant impact (*P* = 0.000***) of the sub‐county on the perception of *infrastructure* in 2018. A significant difference (*P* = 0.010**) between Namisuni (4.24 ± 0.60) and Simu (3.79 ± 1.04) was found, as well as a very high significant difference (*P* = 0.000***) between Simu and Bulegeni (4.26 ± 0.95), but no significant difference between Namisuni and Bulegeni. Farmers in Simu perceived the lowest constraint level for poor infrastructure.

For data collected one season later, the ANOVA resulted in *P* = 0.588 for *poor infrastructure* but in a significant (*P* = 0.030*) impact of sub‐county on the *distance to markets*. The additional pairwise comparison highlighted a significant difference (*P* = 0.037*) between Simu (4.56 ± 0.68) and Namisuni (4.26 ± 0.94), with farmers in Simu perceiving a greater negative impact of *distance to markets*.

The item of *lack of insurance* could not be assigned to one of the identified factors, but the results of this indicator should not be overlooked: Neither time (*P* = 0.682) nor sub‐county showed an impact on the perception of *lack of insurance*, neither in 2018 (*P* = 0.628) nor in 2019 (*P* = 0.292). For perceptions of the *lack of insurance*, no HH gave a qualitative answer for either a perceived improvement or a perceived deterioration.

*Lack of health care nearby* also could not be assigned to any one factor compiled by the PCA. When investigating the perception of *lack of health care nearby*, no significant impact of time (*P* = 0.712) resulted from the *t*‐test for the difference of total mean of 4.13 ± 1.19 in 2018 to 4.18 ± 1.21 in 2019.

While only nine farmers provided detailed answers on a perceived improvement in health care, 39 farmers explained a perceived deterioration of that indicator. Eight of the nine HHs that perceived and specified an improvement in the health care situation mentioned that a “new health facility was established” and that they were now “able to get treatment.” The perception that “the health center has been moved closer to the community,” thereby reducing the distance, was mentioned several times.

The explanation given for deteriorations of the health care item ranged from “a lack of medicine in health centers” to “we lack a health facility entirely.” Many farmers mentioned that the “health center is very far away, yet we lack transport, with poor roads that put our lives at risk,” or in other words, that “the health center is far away, and difficult to access due to bad roads.” Other farmers in the interviewed HHs mentioned that “there is not much help from the government.” One farmer mentioned that the constraint level has increased, not only “due to long distance” but also due to “too much competition.” A resident of Namisuni explained the deterioration of adequate health care by “increased poverty.”

The impact of sub‐county on this indicator was highly significant (*P* = 0.000***) in both years. In 2018, the pairwise comparison showed a very high difference (*P* = 0.000***) between Simu (3.87 ± 1.27) and Bulegeni (4.57 ± 0.73) and Bulegeni and Namisuni (3.87 ± 1.27), but no difference between Simu and Namisuni (*P* = 1.000).

One year later, the difference between sub‐counties was significant for all possible combinations (*P* ≤ 0.05): Namisuni (4.18 ± 1.16) and Simu (3.60 ± 1.51), Simu and Bulegeni (4.51 ± 0.93), and Bulegeni and Namisuni.

For the specification of no other unnamed indicators, two farmers, both located in Simu, mentioned that “harsh drought has affected our crops” and that “the crops were scorched by the sun.”

## 4 Discussion

The results of the survey show that for most items, the mean in constraint levels was above 4 in 2018; only *lack of insurance*, *distance to water source*, and *water quality* showed means below that level. However, for all items except *bad roads*, *water quality*, and *water source*, the standard deviation increased from 2018 to 2019 for the whole sample. In addition, in Bulegeni, the change in standard deviation is weaker in both years. This finding indicates that deficiency perceptions varied more between HHs in 2019 and more within Namisuni and Simu than in Bulegeni or in the year 2018. This suggests that the conditions or resource levels of farms differed more in Bulegeni, which could be explained, for instance, by the lower number of development programs implemented there.

### 4.1 Basic Preconditions for farm Management Activities

#### 4.1.1 Factor 1: Lack of Reliability, Information, and Input Supply

The constraint level for lack of reliable buyers of coffee, lack of reliable sellers of input, and lack of information was perceived as not as bad during the coffee harvest of 2018 as in 2019, both for the entire sample and in each sub‐county. The farmers saw improvements in contracts, reliability of buyers, and access to information because of higher availability of radios, improved communication with other farmers, and access to extension services and groups. This is consistent with findings in Peru, where members of farmers’ organizations had better access to information and markets.^[^ ^5^ ^]^

The lack of knowledge and reliable information in Uganda is well known. According to a study by^[^ ^10^ ^]^ carried out in Southeast Uganda, information networks among farmers, extension workers, local governments, and the private sector are very uncommon, leading to a large information gap. In the present study, farmers were asked to name their three most reliable and desirable sources of information. Other farmers, neighbors, and relatives were mentioned by 288 of the interviewed HHs, followed by the radio, which was mentioned by 274 farmers, and finally by extension staff and offices, which were mentioned by 250 HHs (2019). No significant impact of sub‐county on information sources was found in 2018. The difference between sub‐counties in the item *lack of information* in 2019 could be explained by the different percentages of HHs in each sub‐county using the three most important information sources in 2019: In Bulegeni, only 50 to 62% of the HHs used the individual sources, followed by Simu, where 75 to 86% of the HHs used the sources, and Namisuni, where 83 to 93% of the HHs used the sources of information. These percentages align with the highest constraint level for this indicator in Bulegeni, followed by Simu, and the lowest constraint level in Namisuni.

In all sub‐counties, farmers mentioned that they received more information about coffee (prices) in 2019. This positive trend could be extended further, for instance, by implementing a coffee radio program that could provide scientifically based information about coffee production and how to cope with weather extremes, pests, and diseases (e.g., CBB, CBD), how to implement proper post‐harvest processing methods, what inputs are recommended at what costs, and what coffee prices can be expected for what level of quality. Previous results from^[^ ^17^ ^]^ also indicate that some of the plots in the same districts “showed no or [a] very low coffee productivity as a consequence of old coffee bushes or inappropriate management practices.” Improving farm management practices could therefore also help the Ugandan Coffee Development Authority (UCDA) to reach the goal of quadrupling Uganda's coffee production^[^ ^18^ ^]^ by reducing farmers’ constraint levels for the basic preconditions for farm management and enabling them to increase their coffee productivity. Research on the basic conditions for this could not only help to increase coffee productivity; it could also prevent or slow down the reduction of coffee production because the suitable land for Arabica coffee cultivation in Uganda is declining due to climate change.^[^ ^19^ ^]^

The constraint level for most of the indicators belonging to Factor 1 was lower in Bulegeni in 2018 but rose relative to the other two sub‐counties in 2019, even though there was better access to the farms in Bulegeni from the main road. The higher constraint level in Bulegeni must therefore have been for other reasons than bad roads and low accessibility. A previous study from 2015 investigated the impact of management system and altitude on Arabica coffee quality in the same sub‐counties and found that for all investigated parameters (bean size, bean density, number of defects, cupping score, and weight of 100 beans), coffee quality was lower in Bulegeni than in the other two sub‐counties.^[^ ^20^ ^]^ Due to climate change, the coffee gardens in Bulegeni and other sub‐counties in this altitude range already show lower suitability for Arabica coffee cultivation, which leads to low productivity but also low coffee quality. It could be assumed that during the heavy rainy season of 2019, the buying companies were prioritizing farms that provide higher coffee quality than found in Bulegeni. The additionally higher constraint level in Bulegeni regarding the item *lack of proper machines for field care* could also be explained by altitude and coffee quality. The other questions in the survey also showed higher frustration and lower motivation levels due to the lower welfare and lower liquidity of farmers in Bulegeni, which limits their options and prevents them from improving working conditions for coffee farming. Due to these factors, most outside actors do not focus on supporting farms in Bulegeni, and most machine sellers also prioritize other sub‐counties.

The other major positive impact mentioned was the increased competition between buying companies in the area, which may have forced companies to be more reliable because farmers had more options for selling. At the same time, the number of companies buying coffee was increasing and shares were being divided among smaller stakeholders. The concentration of exporters shows the opposite trend: Ten major exports held 80% of the market share in 2019.^[^ ^21^ ^]^ Returning to the farm gate perspective, in 2018, 715 coffee‐buying stores were registered with the UCDA, and in 2020, the UCDA had 742 coffee‐buying stores registered.^[^ ^15^, ^21^ ^]^ The annual report of UCDA assumes that the competition in the coffee value chain will continue.^[^ ^21^ ^]^ Even though the number of coffee‐buying companies was increasing, from 2018 to 2019 most farmers did not switch to other buyers, nor was there any evidence of a trend toward HHs dividing their products and selling coffee to more than one company.

The reliability of buyers was perceived to have increased because of less cheating on quality standards and weighing scales, which could be explained by the better awareness of coffee quality that was also mentioned. In contrast to information access, here, no difference between sub‐counties was found, so it could be assumed that the improvement in coffee quality awareness did not result from one of the three information sources.

#### 4.1.2 Factor 3: Lack of Prerequisites for Proper Field Care

For the weighing scales, the Ugandan National Bureau of Standards (UNBS) has regulated the standards and now stipulates annual stamping. If calibration and standardization was not carried out at regular intervals, this could be one explanation for the different perceptions of constraint levels in manipulated weighing scales between survey rounds. Another reason could be the trend towards stricter controls and regularities of UNBS. Whether this is a singular improvement or a trend should be investigated further by conducting research over a longer period.

Even though the constraint level for bad roads was very high in 2018, it had nearly reached its peak and showed very low deviation between farms. The reason was the extreme rains of 2019 that destroyed nearly every unpaved road in the region. Also, more than half of the total sample responded to the open‐answer question about *bad roads*, with more than half of the respondents mentioning climate change and heavy rainfalls as the reason for the bad road conditions. This indicates a change in awareness of climate change. In 2015, when we held workshops with 134 farmers in the same sub‐counties, no farmer was aware of climate change or the difference between climate and weather.

The effects of bad roads range from increased transport costs to economic losses due to a failure to transport products to the markets, to inaccessibility of proper health care, to the high risk of life‐threatening accidents. Many farmers also mentioned insufficient support from the government in fixing the roads as a reason for the worse conditions. Those farmers who experienced a deterioration in production explained that the bad roads led to increased costs of transport and reduced options for selling time due to the inaccessibility of markets. This seems to be one of the biggest challenges due to the abruptly changing market prices that already harm output when roads are in better condition.

Although bad roads have severe negative impacts on the farmers’ lives, they also protect them against land‐grabbing. The majority of HHs do not have an official land title, and the nutrient‐rich volcanic soils in the Mount Elgon region attract many companies and investors.^[^ ^22^ ^]^ The only fact that hinders land‐grabbing is the bad roads, which are too difficult to navigate with larger vehicles and machines. As soon as the condition of the roads in the district improves, it is likely that land‐grabbing will take place, and that would have a huge impact on farmers’ lives and wellbeing.^[^ ^7^ ^]^ Mandating official registration of land would be the only means of preventing potential land‐grabbing. Efforts should also be made to establish equal rights between men and women in matrimonial property law. At the moment, it is common practice (although not official) that men hold the land rights in married couples. In the case of the male HH head's death, his male relatives inherit those rights. The loss of land often increases the economic vulnerability of the remaining HH members. This is especially important, considering that about half of the female HH heads in this study are widows.^[^ ^7^ ^]^ Land registration is a bureaucratic and cost‐intensive process. Like coffee certification processes, access to resources, limited cash liquidity, and the educational background required to fill out forms pose obstacles for many farmers. Without outside help, it is unlikely that farmers would know about the risk or be able to register their land officially.

#### 4.1.3 Factor 5: Unfavorable Prices for Inputs and Outputs

Coffee prices vary widely from one year to the next, so the decreasing constraint level on the item of *poor market prices for produce* cannot necessarily be expected to show a clear trend and could simply indicate a singular increase from one year to another. When looking at market prices, it should be further investigated how much was paid for other products that are sold, for instance, at local markets. Previous research showed that the different cultivated products compete with coffee, and that this is highly dependent on coffee prices. If prices are low, farmers tend to produce more local products, some even stump, that is, to cut back some coffee to have more space for other products in the next season, which might bring better prices. High fluctuations in coffee prices make prediction and planning difficult. It can therefore be assumed that, even if prices were perceived as less restrictive in the second round of the survey, the recurring fluctuations and price uncertainties could be one of the main concerns of coffee‐producing households, as is also the case in Mexico, Peru, and other Central American countries.^[^ ^5^, ^23^, ^24^ ^]^

In the long run, however, some experts assume a trend toward better coffee prices due to increasing demand and a reduction in the suitability of land, especially for Arabica coffee cultivation, and thus a reduced supply. Along with the increased competition between buyers that has led to better prices, farmers mentioned that they have developed skills in better storage that allow them to make better decisions about when to sell their produce. This point is important when considering that coffee prices fluctuate not only between seasons but also within the approximately six weeks of harvest per coffee farm, starting earlier at lower altitudes and later at higher altitudes. This is due to the lower quality of coffee before and after the peak of harvest but is also linked to demand and the ability of exporting companies to carry out the processes required after buying and transporting the coffee back to town. Due to many logistical reasons linked to the quantity and quality of coffee available, buyers usually do not buy coffee from the first to the last day possible in a season. This results in coffee prices being the highest around the peak of harvest and the lowest at the beginning and end of the season.

The farmers usually do not have moisture meters or other farm management tools that enable them to measure when to stop drying the coffee. As the coffee loses weight during drying and is paid by the kilogram, farmers are not interested in having coffee that is too dry (which is also harder to sell) and usually stop at a point of uncertainty. This has two drawbacks: first, the coffee could quickly be affected by mold, and second, the weight of the coffee is higher than it would be if properly dried, which means for the buyer that coffee will be more expensive after it is dried further in town. However, farmers mentioned that they had gained skills in proper storage, which could be from training in better estimating the moisture content of coffee based on the visual and haptic qualities of coffee beans. It could further imply storing the coffee dry, in closed bags, on palettes, not next to a wall, far away from animals, and so on. This may have led to greater freedom in decision‐making in selling, which may have impacted the price per kilogram significantly.

No significant differences in perceptions of coffee prices were found between sub‐counties. This could be explained by a difference in quality between sub‐counties that is not large enough to be reflected in the prices, in a huge deviation between supply and demand, or in the quality premiums, which are sometimes paid a few weeks after selling and therefore unlikely to have affected the coffee producers’ perceptions.

However, when looking at the constraint level for inputs, the lower constraint level for the high market prices for inputs found in Namisuni could be explained by a higher number of exporting companies focusing on training and knowledge transfer in that sub‐county due to the higher altitude and better coffee quality expected. Furthermore, Namisuni is closer to Kapchwora, a sub‐county where people are close to the border of Kenya, where they have access to better markets for their products and different conditions for inputs.

Other farmers report that increasing pests and diseases or severe droughts reduce productivity and income from selling. Farmers seem to experience input and output as even more out of balance when their liquidity is low. The lack of liquidity hinders them, on the one hand, from waiting for the best time to sell their products, which reduces their profits. On the other hand, financial obligations such as loans increase the pressure to cover interest and repayment rates. The combination of the two often leads to persistent low liquidity, frustration, and lower motivation and ability to invest in production.

A few farmers also saw a deterioration in the factor of increasing pests and diseases or droughts due to increased poverty resulting in a lack of proper machines and appropriate business practices: They referred to inadequacies in weighing scales as well as a low level of trust in the honesty of buyers. It can be assumed that the farmers mentioned have less access to information or lower freedom to choose buyers due to the location of their farms or their position in the community. It could further be assumed that those farmers experience higher time pressure due to greater economic vulnerability, which could lead to emergencies and also to lower freedom in decision‐making due to the lack of liquidity required, for instance, to save the lives of family members.

Perceived deteriorations were again explained largely by the changing weather conditions and climate change. Along with heavy rains, farmers mentioned that past droughts had reduced productivity.

#### 4.1.4 Exploitative Middlemen

A very high constraint level was found for exploitative middlemen. It is well known that middlemen are often engaged in unfair and exploitative business practices, such as those found in Uganda.^[^ ^23^ ^]^ However, a highly significant decrease in deficiency perceptions for this indicator was found from 2018 to 2019. Farmers cited two main reasons for this trend: first, better information access and awareness of current coffee prices, and second, an increase in buyers and thereby a larger number of companies buying directly at the farm gate. Increasing numbers of companies avoid buying coffee from middlemen due to mixed and lower coffee quality. Differences in quality therefore cannot be reflected in prices. Furthermore, the middlemen usually try to hide information about coffee value and buy the coffee at a lower price. However, due to the increasing number of buyers in the area, the constraint level is reduced. For the buying companies, buying through middlemen means a reduction in quality. Companies are also able to pay quality premiums when they buy their coffee at the farm gate, which could explain the trend toward fewer middlemen. It is relatively unsurprising that the constraint level regarding middlemen was higher in 2018 in Bulegeni than in Simu because fewer companies were buying coffee in Bulegeni. This was linked directly to lower coffee quality and gave middlemen more space for exploitative behavior.

For those farmers who experienced the exploitative middleman becoming worse, it can be assumed that their farms were less accessible to companies, which usually come with big trucks that cannot navigate all roads, or that they had lower access to information on prices than farmers that experienced an improvement.

The research on wellbeing that used the first round of interviews from the same survey, concluded that contracts between buyers and sellers, improved access to information about the coffee market to reduce the information gap between buyers and coffee farmers, reliable weighing scales, or even a statutory minimum price could be approaches to increase income from coffee selling and mitigate issues of mistrust.^[^ ^7^ ^]^ When looking at the total group for the preconditions for farm management activities and the investigated results of data collected one year later, a trend in this direction is evident. Further research should compare the two survey rounds on an economic basis to test the hypothesis that improvements in Factor 1 correlate with income from coffee‐selling activities.^[^ ^7^ ^]^

### 4.2 General Life Quality

#### 4.2.1 Factor 2: Water Supply Consists of Distance to Water Source and Water Quality

In 2018, the factor of *lack of water supply*, comprising the variables *water quality* and *distance to water source*, had the lowest level of constraint both overall and within single sub‐counties. Both indicators showed a highly significant decrease in deficiency perception from 2018 to 2019. Distance and water quality were perceived to be not as bad in the second survey round for the total sample due to the distribution of water taps and greater access to piped water. Even though the average water quality was perceived to be better in the second year, farmers also noted that water quality differs between dry and rainy seasons, which could be explained by the contamination of water with mud in the rainy season but also by contamination with chemicals and excrement during floods. In contrast to the survey on local knowledge conducted by Cerdán et al. in Costa Rica, the farmers surveyed here, did not consider water management and the use of chemicals on their farms to be factors influencing water quality.^[^ ^6^ ^]^ During rainy seasons and floods, water availability was perceived as better, but water quality was perceived to be lower.

The improvement was explained largely by the distribution of water taps and the extension of piped and treated water by the government. Although most farmers perceived an improvement, a few mentioned that the water supply was worse than the year before. The majority of these lived in Bulegeni, where farmers experienced lower water quality, especially in rainy seasons. Bulegeni is, compared to the other investigated sub‐counties, the one with the lowest slope. During intense rains, it is often flooded, which could explain the perceived lower quality of the water, which also leads to higher infection rates. The deficiency level in Bulegeni was also found to be very high and significantly higher than perceptions in Namisuni in 2018. In the second round of the survey, the picture changed slightly: In 2019, both indicators were perceived as not as bad in Namisuni as in the other two sub‐counties at a highly significant level. This could be explained by the fact that Namisuni has a river and a water service that conducts water from the river to the tap. Most HHs have free tap water in Namisuni, and in Simu, they have wells and waterfalls. This could also explain why the constraint level for distance to the water source is not as good as for farmers in Namisuni but still better than in Bulegeni.

It is unsurprising that water quality is perceived to be better in Namisuni than in Simu or Bulegeni, since the issue of flooding due to less slope is greater in Simu and Bulegeni. It can also be assumed that in times of flooding, acceptable water quality affects perceptions of distance to water. Therefore, the same questions should be asked during the dry season, when the water quality is slightly better in general. It can be assumed that the presence of Sissiyi Falls in Simu could provide a more consistent water source for crop cultivation or reduce the impact of droughts. For those parts of the region without waterfalls or rivers, it can be assumed that the distance to the water source is also perceived as a greater constraint in the dry season.

#### 4.2.2 Factor 4: Infrastructure Includes Poor Infrastructure and Distance to Markets

Both indicators, distance to markets and poor infrastructure, were perceived as highly constraining in both survey rounds, 2018 and 2019. Time was only found to affect the reduced constraint level for distance to markets. Concluding from the qualitative responses of farmers that there was a higher level of buying activities at farm gates, the perceived improvement in distance to markets may have resulted from better transportation of products and the possibility to sell the coffee anywhere. The positive impact of these changes on distance to markets appears to have been greater than the possible negative impact of bad roads. However, the impact of the conditions of bad roads is still not very clear; the item *infrastructure* has the lowest constraint level in Simu and is still perceived as very constraining in Bulegeni and Namisuni. A better understanding of these figures could be gained by allowing farmers to define their understanding of infrastructure and explain what they perceive to be included in that wording.

#### 4.2.3 Lack of Insurance and Lack of Health Care Nearby

The items *lack of insurance* and *lack of health care nearby* did not show a significant difference between the seasons. Some farmers saw an improvement in access after construction or improvement of health facilities. Due to long distances and bad roads, traveling to the hospital is linked with high costs, risks that it may take too long in times of acute illness, and difficulties maintaining farm activities and communication if it is necessary to stay in the hospital for more than one night (most farmers do not have electricity to charge their phones, which meant that we were usually not able to call them or their relatives during hospital stays). Furthermore, depending on conditions, it may be so dangerous to travel to the hospital that farmers risk the lives of the sick and those accompanying them. The constraint level was higher in Bulegeni in 2018 and remained the highest there in 2019 as well, but with a significant difference between the three sub‐counties. In 2018, the difference between Bulegeni and the two other sub‐counties could be explained by the lower wellbeing and living standards in Bulegeni, but also by the probably higher occurrence of sickness.^[^ ^7^ ^]^ No or just a small impact of bad road conditions can be assumed for 2018 because the rains in that harvest season were moderate. In the second survey round, the perception of health care around farms in Simu was improving, while farmers in Namisuni perceived the situation as worse than the previous year. In Bulegeni, the situation remained more or less the same (>4.5). Households in Bulegeni had very bad conditions to start with, whereas farmers in Namisuni perceived the lack of health care nearby as having become worse “due to increased poverty and decreased accessibility,” potentially a direct and indirect consequence of the bad roads. In contrast, HHs living in Simu noted the “upgrading of the hospital” and the “establishment of a new health center” and considered these to be reasons for the improved health situation.

Weather conditions in the two survey rounds differed. While the rainy season of 2018 was moderate, the rains of 2019 led to flooding in low altitudes and mudslides on mountain slopes. Most coffee farmers in the area under investigation lost their annual harvests, and many lost their homes and lives, which cannot be without consequences for the perception of deficiencies.

The more extreme weather conditions in the second round of the survey could explain the reduced size of the sample in 2019. Discrepancies in the data, particularly for the gender of the HH head, could not be excluded in the interpretation of the results. We interviewed the HH heads, who in our sample group were mainly men. Considering that, based on their role within the HH, women are more likely to consider the entire family when reporting their perceptions of deficiencies, there might be different priorities among the various indicators. One could assume that the constraint level of the indicators in general life quality group would have been higher if the women in the HHs had been interviewed. A female perspective might also give more attention to health or educational indicators, such as those found in the Women's Capabilities Index for Malawi developed.^[^ ^24^ ^]^ Due to the widespread gendered division of labor in Uganda and the corresponding differences in men's and women's responsibilities for coffee‐related tasks,^[^ ^25^ ^]^ it might be difficult to collect high‐quality data from the HH heads’ wives on questions about the preconditions for farm management or economic data on HH production, realms that are traditionally the husband's responsibility. It should be kept in mind that the gendered divided division of labor also affects some of the constraint levels for general life quality under investigation. The responsibility for the water supply of the HH is in the hands of the women; One can therefore assume that the results would have been different if the women had been asked about the water situation. This issue does not have a major impact on comparability within our sample group, but the higher number of male‐headed HHs interviewed for this study than is usual in this research area reduces the representativeness of the results for the research area as a whole.

The investigation of two survey rounds in this study has proven to be a good approach: The data from more than one season revealed significant differences for most indicators that could not have been found when analyzing data from one harvest season only, and also highlighted the impact of weather conditions on constraint levels. Another strength of the data collected here comes from the qualitative responses on the reasons for the changes in perceptions of the individual deficiency indicators.

Further research should investigate deficiency perceptions over more than two survey rounds to gain a more reliable long‐term perspective for many indicators and to obtain a clearer picture of whether the results show evidence of a trend or a one‐time change due to the different weather conditions perceived. It would also be useful to implement more indicators that might have an impact on perceptions to study these findings in relation to correspondingly investigated single indicators. The distance to health care centers, for instance, may have been impacted significantly by the health status of the interviewed person or his/her family members. Such data were not collected and could not be assessed in the analyses. For farm management‐related data, the case could be similar. Coffee yields, expectations, productivity per tree, pest and disease occurrence, and the existence of required knowledge to cope with given challenges could also have an impact on the constraint level.

## 5 Conclusion

The main aim of this paper was to understand how coffee farmers in the Mount Elgon region of Uganda perceive deficiencies in the conditions for coffee farming. The implemented PCA, ANOVA, and t‐test, as well as the analysis of responses to open‐answer questions, served as effective tools to test the hypotheses that (1) deficiency perception is not equally distributed across sub‐counties or across the two survey rounds in the Mount Elgon region, and that (2) climate change and other externally influenced conditions have an impact on perceived constraint levels. The paper further highlighted the potential impact of information access and road conditions on profits from coffee production and selling activities. It also indicates a very high constraint level and shows how one event could lead to chain reactions in the conditions for farm management activities but also for general life quality. The heavy rainfalls experienced in 2019 are a good example. With regard to the conditions required for farm management activities, these rains led to nearly impassable roads, more expensive and dangerous transportation of products, fewer choices about time of selling, lower coffee prices but higher input prices, and ultimately, lower output from coffee production. On the other hand, the heavy rainfall also caused mudslides and flooding of houses and toilets, which in turn led to homelessness, loss of harvests, higher infection rates, difficulties transporting sick and injured people to hospitals, and ultimately the loss of life and an overall decrease in life quality. It was also found that worse‐case scenarios are more likely with more vulnerable HHs. Based on these findings, I suggest that further research should be implemented to focus on how the HHs experience shocks and how they react to identify strategies for avoiding worse‐case scenarios and chain reactions.

However, it should be noted that the perceived increase in the price of products and the reduction in input costs was largely explained by improved skills, knowledge, and access to information on prices. Farmers now tend to store their products properly until they are satisfied with prices, which often fluctuate widely even during a single season, and use information from extension services to produce organic fertilizers instead of buying expensive chemicals. Due to the substantial positive impact of better access to information on several of the deficiencies identified, one recommendation would be to implement a verified open‐source information system, for instance, a radio program that provides verified information on coffee prices, pests, and diseases or suitable inputs.

This paper also found that decision making has not only been improved by better skills of farmers; farmers’ choices have been increased quantitatively and qualitatively through market development and increased competition between buyers. At the same time, farmers experienced a limitation of their output due to the bad roads, which led to increased costs of transport and a reduced choice of time to sell due to inaccessibility of markets. This seems to be one of the biggest challenges in light of abruptly changing market prices that have a negative impact on output even when roads are in better condition. The newly achieved advantage of better coffee quality awareness and better levels of information on storage and price fluctuations is thus severely limited by the weather‐dependent ability to move due to intense rains and impassable roads. Increasing pests and diseases or severe drought also have a major impact on productivity and income from coffee production. Along with less suitable weather conditions and consequences of climate change, the imbalance between input and output seem even greater when farmers lack liquidity. On the one hand, this lack hinders them from waiting for the best time to sell their products, which reduces their profit margin. On the other hand, financial obligations, for instance, loans increase the pressure to cover interest and repayment rates. The combination of both often leads to persistent low liquidity, frustration, and lower motivation and ability to invest in production.

While for most of the conditions mentioned, bad road conditions make the situation worse, the bad roads also protect farmers from land‐grabbing. One recommendation could therefore be, in a first stage, to provide help to farmers to obtain official land titles, and in a second stage, to improve the conditions of the roads. The resulting advantages of better information access, competition between buyers, skills, know‐how, and coffee awareness could lead to a better income situation for the coffee‐producing HHs. The tools identified here are therefore not only important to improve the wellbeing of the coffee farmers; they also provide a sound basis for recommendations on how non‐governmental organizations can better address limitations resulting from improper field management conditions. This is especially relevant when considering two points: 1) the goal of UCDA to quadruple Uganda's coffee production by 2040,^[^ ^18^ ^]^ and 2) the increasing challenges resulting from climate change and its impact on general quality of life but also the basic conditions required for farm management.

Although the results for the geographic area investigated here are not directly applicable to other areas, some deficiencies are similar in coffee cultivation areas around the globe. Many coffee‐producing communities are facing extreme weather conditions, such as droughts or heavy rainfalls and impassable roads, more and new pests and diseases, knowledge gaps, limited information access, and poverty. Here, for all indicators, changes were mainly perceived to result from external forces. Deterioration was mainly explained by perceived changes in weather conditions that are caused by climate change. Activities and services brought into communities from the outside were mentioned as major reasons for improvements. The access to material resources such as water taps but also to intellectual resources such as knowledge that was brought to the farms in the form of training, improved information access, and increased competition between buyers improved the situation for farmers, resulting in less opportunistic behavior and better quality and price awareness. This is also very likely to be found in research in other coffee cultivation areas.

In conclusion, the results presented here suggest that, on the one hand, the already high constraint level of the HHs in this area will continue to increase in the future due to climate change and its direct and indirect impacts on life quality and the basic conditions required for farm management. On the other hand, this study also found that the greater competition between coffee buying companies and activities or resources brought into these regions from the outside to better cope with challenges are having a positive impact on farmers’ physical and mental health. This paper suggests that if these activities—especially the expansion of information access and improvement of road conditions (after land registration)—are implemented on a larger scale, the negative trends caused by deteriorating weather and accelerating climate change could be (partly) counteracted to help farmers maintain the conditions for effective farm management and improve their quality of life in the future.

## Conflict of Interest

The authors declare no conflict of interest.

## Supporting information

Supporting Information

## Data Availability

The data that support the findings of this study are available from the corresponding author upon reasonable request.
