# Supplementary material for: Perceptions of Deficiencies in the Basic Conditions for Farm Management and Quality of Life in Coffee‐Growing Households: A Panel Analysis of a Rural Community in Eastern Uganda
Source: Glob Chall. 2024 Mar 12;8(4):2300196. doi: 10.1002/gch2.202300196 (PMC11009422; doi:10.1002/gch2.202300196)
Supplement: Supplementary file 1 — Supporting Information [file GCH2-8-2300196-s001.pdf]

Global Challenges
Open Access

Supporting Information

for *Global Challenges.*, DOI 10.1002/gch2.202300196

Perceptions of Deficiencies in the Basic Conditions for Farm Management and Quality of Life in Coffee-Growing Households: A Panel Analysis of a Rural Community in Eastern Uganda

*Anna Lina Bartl**

Supporting Information

Perceptions of deficiencies in the basic conditions for farm management and quality of life in coffee-growing households: A panel analysis of a rural community in Eastern Uganda

Anna Lina Bartl

Table S1. Mean and standard deviation for the five identified factors

Factor		Bulegeni (n=134)		Simu (n=77)		Namisuni (n=149)		Total (n=360)	
		**2018**	**2019**	**2018**	**2019**	**2018**	**2019**	**2018**	**2019**
**Conditions for farm management activities**									
Factor 1: Reliability	Mean	4.47	4.11	4.26	4.09	4.37	3.88	4.12	3.01
	SD	0.79	0.89	0.66	0.94	0.64	1.08	0.69	1.58
Factor 3: Prerequisite	Mean	3.87	3.45	4.32	3.41	4.27	2.96	4.51	4.27
	SD	1.17	1.16	0.66	1.10	0.59	1.17	0.45	0.60
Factor 5: Prices	Mean	3.76	3.50	4.28	3.23	4.27	2.97	4.43	3.88
	SD	1.24	1.08	0.65	1.09	0.59	1.14	0.54	0.76
**General life quality**									
Factor 2: Water supply	Mean	3.58	3.65	3.75	3.94	3.70	3.66	3.01	2.77
	SD	1.44	1.47	1.23	1.22	1.15	1.40	1.58	1.33
Factor 4: Infrastructure	Mean	4.57	4.51	3.87	3.60	3.87	4.18	4.31	4.28
	SD	0.73	0.93	1.43	1.51	1.27	1.16	0.65	0.71

*Scale from 1 (=constrains me not at all) to 5 (=constrains me very much).

Table S2. Paired t-test to measure the impact of time on single deficiency indicators

	Difference in Mean±SE	Paired differences, SE of the mean	95% confidence interval, lower	95% confidence interval, upper	T	df	P>T
**Conditions for farm management activities**							
Exploitative middleman	0.380±1.247	0.066	0.249	0.510	5.721	352	**0.000*** **
Lack of contracts or reliable buyers	0.895±1.435	0.076	0.745	1.045	11.718	352	**0.000*** **
Lack of contract or reliable sellers	0.861±1.470	0.078	0.707	1.015	11.010	352	**0.000*** **
Lack of information	1.159±1.496	0.080	1.002	1.316	14.541	351	**0.000*** **
Lack of input supply	0.736±1.344	0.072	0.594	0.879	10.178	344	**0.000*** **
Bad roads	-0.209±0.889	0.048	-0.302	-0.115	-4.390	349	**0.000*** **
Lack of proper machines for field management	0.313±1.105	0.060	0.196	0.430	5.261	344	**0.000*** **
Cheating on quality standards/weighing scales	0.629±1.444	0.077	0.478	0.780	8.180	352	**0.000*** **
Poor market prices produce	0.506±1.076	0.057	0.393	0.618	8.866	355	**0.000*** **
High market for inputs	0.596±1.184	0.063	0.472	0.720	9.468	353	**0.000*** **
**General life quality**							
Lack of insurance	-0.043±1.968	0.106	-0.252	0.165	-0.410	344	0.682
Lack of health care near by	-0.031±1.588	0.085	-0.198	0.135	-0.369	351	0.712
Distance to water source	0.384±2.264	0.121	0.146	0.621	3.178	351	**0.002** **
Water quality	0.068±2.279	0.122	-0.171	0.308	0.562	350	0.574
Poor infrastructure	-0.045±1.250	0.066	-0.176	0.085	-0.680	353	0.497
Distance markets	0.107±1.153	0.061	-0.013	0.227	1.747	355	0.082[1]

Table S3. Paired T-test to measure the influence of time on the five identified factors

	Difference in Mean ± SE	Paired difference, standard error of the mean	95% confidence interval of the difference, lower	95% confidence interval of the difference, upper	T	df	P>T
**Conditions for farm management activities**							
Factor 1: Reliability	-0.077±1.460	0.085	-0.245	0.091	-0.905	292	0.366
Factor 3: Prerequisite	0.020±1.324	0.077	-0.132	0.173	0.264	292	0.792
Factor 5: Prices	0.026±1.382	0.081	-0.133	0.185	0.321	292	0.748
**General life quality**							
Factor 2: Water supply	0.021±1.277	0.075	-0.126	0.168	0.285	292	0.776
Factor 4: Infrastructure	0.028±1.340	0.078	-0.126	0.182	0.354	292	0.723

[1] For distance to markets, the Wilcoxon Test is showing a significant result (P=0,020*).

Table S4. One-factor ANOVA for the influence of sub-county on the variables in 2018

2018		Square sum	df	Mean of the squares	F	Significance
**Conditions for farm management activities**						
Exploitative middleman	Between Groups	2.189	2	1.095	2.191	0.113[2]
	Within Groups	176.317	353	0.499		
	Total	178.506	355			
Lack of reliable buyers	Between Groups	14.155	2	7.077	9.467	0.000***
	Within Groups	263.901	353	0.748		
	Total	278.056	355			
Lack of reliable sellers	Between Groups	21.647	2	10.823	13.446	0.000***
	Within Groups	284.151	353	0.805		
	Total	305.798	355			
Lack of information	Between Groups	0.289	2	0.144	0.186	0.830
	Within Groups	273.204	352	0.776		
	Total	273.493	354			
Lack of input supply	Between Groups	1.327	2	0.664	0.793	0.453
	Within Groups	293.013	350	0.837		
	Total	294.340	352			
Bad roads	Between Groups	0.488	2	0.244	0.460	0.632
	Within Groups	185.620	350	0.530		
	Total	186.108	352			
Lack of proper machines for field care	Between Groups	0.501	2	0.251	0.443	0.643
	Within Groups	198.139	350	0.566		
	Total	198.640	352			
Cheating on quality standards/weighing scales	Between Groups	0.295	2	0.148	0.464	0.629
	Within Groups	112.342	353	0.318		
	Total	112.638	355			
Poor market prices for produce	Between Groups	0.913	2	0.456	1.063	0.346
	Within Groups	151.559	353	0.429		
	Total	152.472	355			
High market prices for inputs	Between Groups	0.848	2	0.424	0.977	0.377
	Within Groups	153.138	353	0.434		
	Total	153.986	355			
**General life quality**						
Lack of insurance	Between Groups	1.525	2	0.762	0.466	0.628
	Within Groups	570.586	349	1.635		
	Total	572.111	351			
Lack of health care nearby	Between Groups	40.994	2	20.497	15.761	0.000***
	Within Groups	457.783	352	1.301		
	Total	498.777	354			
Distance to water source	Between Groups	12.132	2	6.066	2.068	0.128
	Within Groups	1032.504	352	2.933		
	Total	1044.637	354			
Water quality	Between Groups	45.043	2	22.521	8.265	0.000***
	Within Groups	953.683	350	2.725		
	Total	998.725	352			
Poor infrastructure	Between Groups	12.547	2	6.273	8.814	0.000***
	Within Groups	250.541	352	0.712		
	Total	263.087	354			
Distance to markets	Between Groups	2.095	2	1.048	1.791	0.168
	Within Groups	206.500	353	0.585		
	Total	208.596	355			

[2] Kruskal-Wallis Test showed 0,007*.

Table S5. Pairwise comparison 2018 to identify the reason for significant impact of sub-county on perception of deficiencies found in ANOVA

**Indicators 2018**	**Sub-county**	**Test statistics**	**Standard error**	**Standard test statistics**	**Significance**	**Corr. Significance**
**Conditions for farm management activities**						
Exploitative middleman	Simu-Namisuni	17.175	12.935	1.328	0.184	0.553
	Simu-Bulegeni	40.081	13.165	3.044	**0.002****	**0.007****
	Namisuni-Bulegeni	22.906	10.957	2.091	**0.037***	0.110
Lack of reliable buyers	Simu-Namisuni	-9.414	13.344	-0.706	0.480	1.000
	Simu-Bulegeni	-30.854	13.581	-2.272	**0.023***	0.069
	Namisuni-Bulegeni	-21.440	11.303	-1.897	0.058	0.174
Lack of reliable sellers	Simu-Namisuni	-3.466	13.320	-0.260	0.795	1.000
	Simu-Bulegeni	-33.679	13.556	-2.484	**0.013***	**0.039***
	Namisuni-Bulegeni	-30.213	11.282	-2.678	**0.007****	**0.022***
**General life quality**						
Lack of health care nearby	Simu-Namisuni	-9.942	13.235	-0.751	0.453	1.000
	Simu-Bulegeni	51.829	13.455	3.852	**0.000*****	**0.000*****
	Namisuni-Bulegeni	61.771	11.216	5.507	**0.000*****	**0.000*****
Water quality	Simu-Namisuni	-16.662	13.849	-1.203	0.229	0.687
	Simu-Bulegeni	29.781	14.132	2.107	**0.035***	0.105
	Namisuni-Bulegeni	46.443	11.726	3.961	**0.000*****	**0.000*****
Poor infrastructure	Simu-Namisuni	37.873	12.903	2.935	**0.003****	**0.010****
	Simu-Bulegeni	56.093	13.150	4.266	**0.000*****	**0.000*****
	Namisuni-Bulegeni	18.220	10.951	1.664	0.096	0.288

Table S6. One-factor ANOVA for the influence of sub-county on the variables in 2019

		Square sum	df	Mean of the squares	F	Significance
**Conditions for farm management activities**						
Exploitative middleman	Between Groups	4.277	2	2.139	2.215	0.111
	Within Groups	342.698	355	0.965		
	Total	346.975	357			
Lack of reliable buyers	Between Groups	19.761	2	9.880	7.446	0.001***
	Within Groups	471.058	355	1.327		
	Total	490.818	357			
Lack of reliable sellers	Between Groups	19.528	2	9.764	7.959	0.000***
	Within Groups	435.478	355	1.227		
	Total	455.006	357			
Lack of information	Between Groups	42.990	2	21.495	13.512	0.000***
	Within Groups	564.730	355	1.591		
	Total	607.721	357			
Lack of input supply	Between Groups	22.272	2	11.136	13.740	0.000***
	Within Groups	283.677	350	0.811		
	Total	305.949	352			
Bad roads	Between Groups	5.949	2	2.975	10.162	0.000***
	Within Groups	103.917	355	.293		
	Total	109.866	357			
Lack of proper machines for field care	Between Groups	3.151	2	1.575	2.505	0.083
	Within Groups	220.079	350	0.629		
	Total	223.229	352			
Cheating on quality standards/weighing scales	Between Groups	2.229	2	1.115	0.627	0.535
	Within Groups	630.866	355	1.777		
	Total	633.095	357			
Poor market prices for produce	Between Groups	2.759	2	1.379	1.979	0.140
	Within Groups	249.579	358	0.697		
	Total	252.338	360			
High market prices for inputs	Between Groups	6.638	2	3.319	3.590	0.029*
	Within Groups	329.117	356	0.924		
	Total	335.755	358			
**General life quality**						
Lack of insurance	Between Groups	4.773	2	2.386	1.234	0.292
	Within Groups	678.541	351	1.933		
	Total	683.314	353			
Lack of health care near by	Between Groups	40.394	2	20.197	14.767	0.000***
	Within Groups	485.520	355	1.368		
	Total	525.913	357			
Distance to water source	Between Groups	65.031	2	32.515	13.920	0.000***
	Within Groups	829.237	355	2.336		
	Total	894.268	357			
Water quality	Between Groups	38.673	2	19.336	8.631	0.000***
	Within Groups	795.316	355	2.240		
	Total	833.989	357			
Poor infrastructure	Between Groups	0.791	2	0.395	0.531	0.588
	Within Groups	265.598	357	0.744		
	Total	266.389	359			
Distance to markets	Between Groups	4.722	2	2.361	3.553	0.030*
	Within Groups	237.893	358	0.665		
	Total	242.615	360			

Table S7. Pairwise comparison 2019 to identify the reason for significant impact of sub-county on perception of deficiencies found in ANOVA

Indicators	Sub-county	Test statistics	SE	Standard test statistics	Significance	Corr. Significance
**Conditions for farm management activities**						
Lack of reliable buyers	Simu-Namisuni	-37.224	13.960	-2.667	0.008**	0.023*
	Simu-Bulegeni	5.796	14.285	0.406	0.685	1.000
	Namisuni-Bulegeni	43.020	11.963	3.596	0.000***	0.001***
Lack of reliable sellers	Simu-Namisuni	-21.788	13.918	-1.565	0.117	0.352
	Simu-Bulegeni	27.798	14.229	1.954	0.051	0.152
	Namisuni-Bulegeni	49.586	11.982	4.138	0.000***	0.000***
Lack of information	Simu-Namisuni	-25.954	14.124	-1.838	0.066	0.198
	Simu-Bulegeni	34.791	14.431	2.411	0.016*	0.048*
	Namisuni-Bulegeni	60.745	12.029	5.050	0.000***	0.000***
Lack of input supply	Simu-Namisuni	-26.055	13.553	-1.922	0.055	0.164
	Simu-Bulegeni	33.330	13.861	2.405	0.016*	0.049*
	Namisuni-Bulegeni	59.385	11.677	5.086	0.000***	0.000***
Bad roads	Simu-Namisuni	-0.120	9.289	-0.013	0.990	1.000
	Simu-Bulegeni	-37.519	9.497	-3.951	0.000***	0.000***
	Namisuni-Bulegeni	-37.399	7.997	-4.676	0.000***	0.000***
Lack of proper machines for field care	Simu-Namisuni	-2.913	12.607	-0.231	0.817	1.000
	Simu-Bulegeni	28.802	12.962	2.222	0.026*	0.079
	Namisuni-Bulegeni	31.715	10.899	2.910	0.004**	0.011**
High market prices for inputs	Simu-Namisuni	-30.028	13.657	-2.199	0.028*	0.084
	Simu-Bulegeni	-2.973	13.920	-0.214	0.831	1.000
	Namisuni-Bulegeni	27.055	11.662	2.320	0.020*	0.061
**General life quality**						
Lack of health care near by	Simu-Namisuni	37.204	12.931	2.877	0.004**	0.012*
	Simu-Bulegeni	64.913	13.233	4.905	0.000***	0.000***
	Namisuni-Bulegeni	27.709	11.082	2.500	0.012*	0.037*
Distance to water source	Simu-Namisuni	-50.958	13.810	-3.690	0.000***	0.001***
	Simu-Bulegeni	7.913	14.118	0.560	0.575	1.000
	Namisuni-Bulegeni	58.871	11.889	4.952	0.000***	0.000***
Water quality	Simu-Namisuni	-54.060	14.076	-3.840	0.000***	0.000***
	Simu-Bulegeni	-19.238	14.353	-1.340	0.180	0.540
	Namisuni-Bulegeni	34.822	12.099	2.878	0.004**	0.012**
Distance to markets	Simu-Namisuni	-32.739	13.084	-2.502	0.012*	0.037*
	Simu-Bulegeni	-29.479	13.354	-2.208	0.027*	0.082
	Namisuni-Bulegeni	3.261	11.215	0.291	0.771	1.000

Each row tests the null hypothesis that the distributions in sample 1 and sample 2 are the same. Asymptotic significances (two-sided tests) are shown. The significance level is .050. Significance values are adjusted by the Bonferroni correction for multiple tests.

Table S8. One-factor ANOVA for the influence of sub-county on the identified factors for 2018

2018	Source	Partial SS	df	MS	F	P(>F)
**Conditions for farm management activities**						
Factor 1: Reliability	Between Groups	14.810	2	7.405	7.699	0.001***[3]
	Within Groups	322.190	335	0.962		
	Total	337.000	337			
Factor 3: Prerequisite	Between Groups	22.998	2	11.499	12.268	0.000***
	Within Groups	314.002	335	0.937		
	Total	337.000	337			
Factor 5: Prices	Between Groups	1.146	2	0.573	0.572	0.565
	Within Groups	335.854	335	1.003		
	Total	337.000	337			
**General life quality**						
Factor 2: Water supply	Between Groups	14.405	2	7.203	7.480	0.001***
	Within Groups	322.595	335	0.963		
	Total	337.000	337			
Factor 4: Infrastructure	Between Groups	1.994	2	0.997	0.997	0.370
	Within Groups	335.006	335	1.000		
	Total	337.000	337			

Table S9. Pairwise comparison 2018 to identify the reason for significant impact of sub-county on perception of deficiencies found in ANOVA

2018	Sub-county	Test statistics	SE	Standard test statistics	Significance	Corr. Significance
**Conditions for farm management activities**						
Factor 3: Prerequisite	Simu-Namisuni	38.743	14.073	2.753	0.006**	0.018*
	Simu-Bulegeni	74.790	14.437	5.180	0.000***	0.000***
	Namisuni-Bulegeni	36.047	12.036	2.995	0.003**	0.008**
**General life quality**						
Factor 2: Water supply	Simu-Namisuni	-25.936	14.073	-1.843	0.065	0.196
	Simu-Bulegeni	18.536	14.437	1.284	0.199	0.597
	Namisuni-Bulegeni	44.472	12.036	3.695	0.000***	0.001***

Each row tests the null hypothesis that the distributions in sample 1 and sample 2 are the same. Asymptotic significances (two-sided tests) are shown.
The significance level is .050. Significance values are adjusted by the Bonferroni correction for multiple tests.

[3] Kruskall Wallis showed 0,090*.

Table S10. ANOVA for the influence of sub-county on the identified factors for 2019

2019	Source	Partial SS	df	MS	F	P(>F)
**Conditions for farm management activities**						
Factor 1: Reliability	Between Groups	0.539	2	0.270	0.268	0.765
	Within Groups	313.461	312	1.005		
	Total	314.000	314			
Factor 3: Prerequisite	Between Groups	16.909	2	8.455	8.879	0.000***
	Within Groups	297.091	312	0.952		
	Total	314.000	314			
Factor 5: Prices	Between Groups	9.021	2	4.511	4.615	0.011*
	Within Groups	304.979	312	0.977		
	Total	314.000	314			
**General life quality**						
Factor 2: Water supply	Between Groups	8.886	2	4.443	4.543	0.011*
	Within Groups	305.114	312	0.978		
	Total	314.000	314			
Factor 4: Infrastructure	Between Groups	27.274	2	13.637	14.839	0.000***
	Within Groups	286.726	312	0.919		
	Total	314.000	314			

Table S11. Pairwise comparison for the factors that showed a significant impact of sub-county in 2019

2019	Sub-county	Test statistics	SE	Standard test statistics	Significance	Corr. Significance
**Conditions for farm management activities**						
Factor 3: Prerequisite	Simu-Namisuni	-8.888	13.308	-0.668	0.504	1.000
	Simu-Bulegeni	37.076	13.831	2.681	0.007**	0.022*
	Namisuni-Bulegeni	45.964	11.748	3.913	0.000***	0.000***
Factor 5: Prices	Simu-Namisuni	-18.044	13.308	-1.356	0.175	0.525
	Simu-Bulegeni	17.386	13.831	1.257	0.209	0.626
	Namisuni-Bulegeni	35.430	11.748	3.016	0.003**	0.008**
**General life quality**						
Factor 2: Water supply	Simu-Namisuni	-27.001	13.308	-2.029	0.042*	0.127
	Simu-Bulegeni	-1.304	13.831	-0.094	0.925	1.000
	Namisuni-Bulegeni	25.696	11.748	2.187	0.029*	0.086
Factor 4: Infrastructure	Simu-Namisuni	-57.698	13.308	-4.335	0.000***	0.000***
	Simu-Bulegeni	-3.344	13.831	-0.242	0.809	1.000
	Namisuni-Bulegeni	54.354	11.748	4.627	0.000***	0.000***

Each row tests the null hypothesis that the distributions in sample 1 and sample 2 are the same. Asymptotic significances (two-sided tests) are shown. The significance level is .050. Significance values are adjusted by the Bonferroni correction for multiple tests.

Table S12. One-way ANOVA with repeated measurements/Friedmann-Test for the influence of sub-county and time

Source	Type III Sum of Squares	df	MS	F	(P > F)	η^2	Decentr. parameter	Observed discriminatory power
**Conditions for farm management activities**								
**Factor 1: Reliability**								
Year	0.556	1	0.556	0.532	0.466	0.002	0.532	0.112
Year * Sub-county	7.944	2	3.972	3.799	**0.024***	0.026	7.597	0.689
Error (Year)	303.230	290	1.046					
**Factor 3: Prerequisite**								
Year	0.110	1	0.110	0.127	0.721	0.000	0.127	0.065
Year * Sub-county	5.177	2	2.588	2.993	0.052	0.020	5.986	0.578
Error (Year)	250.790	290	0.865					
**Factor 5: Prices**								
Year	0.028	1	0.028	0.030	0.863	0.000	0.030	0.053
Year * Sub-county	5.613	2	2.806	2.978	0.052	0.020	5.956	0.576
Error (Year)	273.299	290	0.942					
**General life quality**								
**Factor 2: Water supply**								
Year	0.019	1	0.019	0.023	0.879	0.000	0.023	0.053
Year * Sub-county	0.430	2	0.215	0.262	0.770	0.002	0.524	0.091
Error (Year)	237.669	290	0.820					
**Factor 4: Infrastructure**								
Year	0.053	1	0.053	0.061	0.806	0.000	0.061	0.057
Year * Sub-county	7.158	2	3.579	4.072	**0.018***	0.027	8.144	0.721
Error (Year)	254.893	290	0.879					
